# Assessing youth-friendly-health-services and supporting planning in the Republic of Moldova

**DOI:** 10.1186/s12978-015-0088-6

**Published:** 2015-10-30

**Authors:** Susanne Carai, Stela Bivol, Venkatraman Chandra-Mouli

**Affiliations:** Schmiljanstrasse 22, 12161 Berlin, Germany; Chișinău, Republic of Moldova; Department of Reproductive Health and Research, World Health Organization, Geneva, Switzerland

## Abstract

**Introduction:**

Several countries have set up youth-friendly-health-services. Relatively little is known about approaches to systematically assess their performance against set standards in terms of quality and coverage and define improvement activities based on the findings. The objective of this paper is to fill this gap and to describe the methods and findings of an external review of youth-friendly-health-services in Moldova and the use of the findings to support further planning.

**Background:**

The Republic of Moldova scaled up youth-friendly-health-services (YFHS) nationwide with the target of setting up at least one youth-friendly-health-centre (YFHC) in each of the 35 districts.

**Methods:**

We carried out an external review of the YFHS in Moldova using a framework that examined the project’s design, implementation and monitoring, outputs, outcomes and impact. We collected primary data - obtained from health worker and client exit interviews with semi-structured questionnaires, direct observation and focus group discussions - and used secondary data from progress reports, previous studies and surveys and national level data.

**Results:**

While impressive progress with geographical scale up had taken place, services were not always provided to the required quality and comprehensively in the newly established YFHC, thereby diminishing chances of achieving the desired outcomes and impact. The causes of this were identified, and possible ways of addressing them were proposed.

**Discussion:**

Designating health facilities to be made youth friendly and assigning health workers to manage them can be done fairly quickly, improving performance takes time and effort. Approaches that go beyond training such as collaborative learning and job shadowing may hold the best opportunity to improve the knowledge, understanding and motivation of health workers in the newly designated YFHC to address the problem of poor quality.

**Conclusions:**

The Healthy Generation project was well designed and energetically implemented in line with the plan. It has contributed to tangible improvements in the quality of health service provision, and to their uptake. While progress has been made, considerable work is needed, especially in the newer centres. If the efforts of the Healthy Generation project are stepped up, if weaknesses in its planning and implementation are addressed, if complementary activities to build knowledge, understanding, skills and an enabling environment are carried out, the project can be expected to improve the health and well- being of Moldova’s young people.

## Introduction

This paper describes an external review of a four-year initiative to scale up the provision of youth friendly health services in Moldova, and the use of the review’s findings to inform the next phase of the initiative. While there are number of reports of evaluations of the quality of health service provision to adolescents and of health service-utilization by adolescents and young people, there are few which systematically assess health centre performance against well-defined standards of quality and against coverage targets, and propose remedial and ameliorative activities based on the findings. The objective of this paper is to fill this gap and to propose a generic evaluation framework that may serve as a template to be used in other settings.

## Background

In 2005 the Ministry of Health of the Republic of Moldova decided to invest in adolescent and young people’s health in order to mitigate the consequences of the breakdown of the Soviet Union and the ensuing social and economic crisis on young people’s health. Moldova’s young people aged 10–24 years constitute almost a quarter of the country’s population of 3.5 million. They have suffered from an increase in deaths from injuries, trauma (including self-inflicted trauma) and intoxications, levels of Sexually Transmitted Infections (STI) including Human Immuno-deficiency Virus (HIV), early and unwanted pregnancies and mental difficulties and disorders [1]. The situation has been aggravated by the on-going massive emigration of the workforce, particularly among the rural population, leaving children and adolescents without parental care. The rate of children and adolescents left behind without parental care in Moldova features among the highest rates in the region [2]. Details of the political, social and economic context and the multifaceted effort of the Ministry of Moldova to respond to the needs of young people effectively have been described elsewhere [1, 3].

**Fig. 1:**
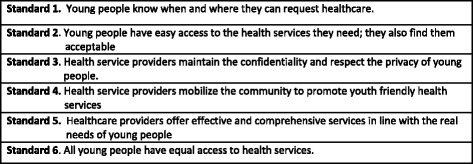
Quality standards for youth friendly health services in the Republic of Moldova [5]

Within the context of a multifaceted effort, Moldova started to set up youth-friendly health services (YFHS) in 2001, with an initial establishment of 3 Youth Friendly Health Centres (YFHC), and then 12 YFHCs in 2005 [4]. In 2009, the MOH developed standards (see Fig. 1) specifying the required quality of services and defined a Package of Services [5] to be provided at each of the youth-friendly health centres including basic and specialized services to prevent and respond to developmental problems, nutritional problems, sexual and reproductive health problems, mental health problems and problems resulting from violence. While all young people were targeted, special efforts were to be made to reach young people who are particularly vulnerable e.g., those living on the streets and those – especially from the rural areas - left behind by parents who have gone abroad.

In 2011, a process of scaling up the YFHS based on WHO’s *systematic approach to improve quality of health services for adolescents* was initiated [6–8]. The approach is based on the WHO-ExpandNet Framework that provides a series of recommendations for programme planning and management, in order to successfully scale-up programmes. According to the Framework, success of the scaling up strategy is determined by multiple interacting factors. Effectiveness of a scaling up strategy depends on characteristics of innovation to be scaled up, as well as characteristics of the resource team and the user organization, each of which is influenced by the environment in which they operate. Successful management of the scaling-up process requires attention to four strategic choice areas - dissemination and advocacy, organizational processes, resource mobilization, and monitoring and evaluation.

**Fig. 2:**
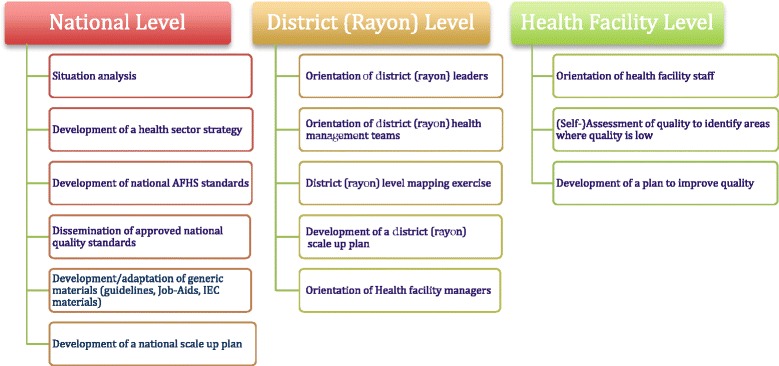
Systematic process for scaling up health sector services provision to adolescents and young people in Moldova

The systematic approach to scale up (Fig. 2) was implemented under the Project Healthy Generation being executed by the non-government organization (NGO) “Health for Youth” (HFY) with support from UNICEF and financed by the Swiss Agency for Development and Cooperation (SDC).

The strategies of the *Healthy Generation Project* involved horizontal and vertical scale up.

Horizontal scale up included:

1. Establishing a YFHC in each district and
2. Building the capacity of health care providers from YFHCs and primary health care services in adolescent health and quality improvement approaches

Vertical scale up included:

(3) Improving the regulatory basis for YFHS
(4) Improving financing mechanisms
(5) Improving the monitoring and evaluation (M&E) system and
(6) Revising the medical university curricula to include adolescent health care.

A nationwide scale-up of youth-friendly-health-services with at least one youth-friendly-health-centre in each of the 35 districts responsible for delivering the required package of health services and meeting the six defined standards of quality was achieved in 2013 with 38 YFHC in 35 districts guaranteeing geographical access to services.

The SDC commissioned an external review at the end of Phase 1 (2011–2014) of the Healthy Generation Project to assess progress and achievements and to draw lessons to inform planning and identify priorities for the second phase.

This paper describes the methods and findings of the external review, which set out to answer the following questions (“if-then” or causal relationships):

- Inputs: Have the project’s plan and budget the potential to achieve the desired outcomes and have the desired impact? Was sustainability built into the projects design?
- Process: Were the activities implemented according to plan?
- Outputs: Were the implemented activities delivered with sufficient quality/coverage to achieve the desired outcomes and have the desired impact?
- Outcome: Have the implemented activities led to the desired outcomes e.g., better knowledge and behaviour changes of young people?
- Impact: Have the achieved outcomes had the desired impact e.g., reduced adolescent pregnancies?
- Cost: Were available resources used efficiently? Are the services sustainable?

## Methods

**Fig. 3:**
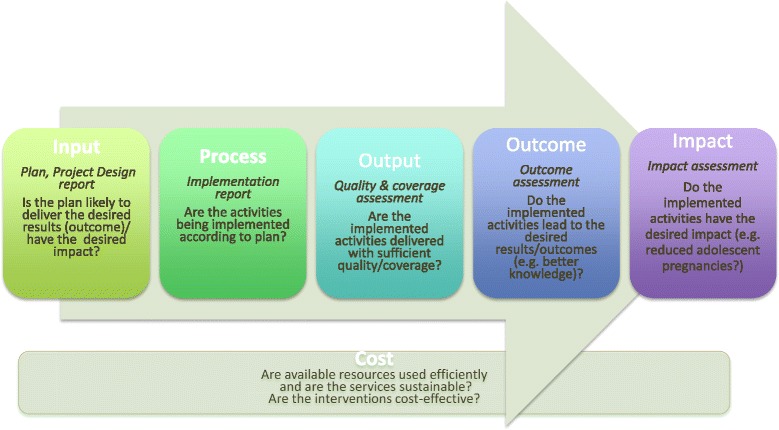
Evaluation framework to assess youth-friendly-health-services (YFHS)

We used the framework shown in Fig. 3 to assess the logical relationships between inputs, processes, outputs, outcomes, and impact, and the cost of YFHS.

**Fig. 4:**
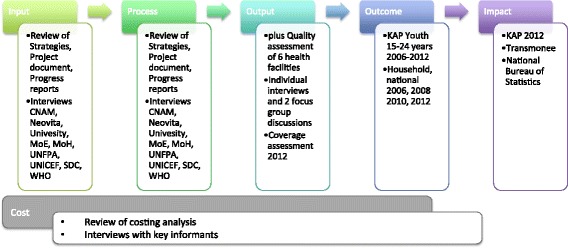
Methods used for the evaluation by area

Methods used to enquire into these questions are summarized in Fig. 4 and included a desk review of relevant national strategies and project documents, progress reports and other published and unpublished documents on YFHS in Moldova. Key informant interviews were carried out with staff of the National Health Insurance CNAM (*Compania Naţională de Asigurări în Medicină*), the NGO *Health for Youth* and Neovita (the NGO and the resource centre providing YFHS at Chisinau), the Medical University, the Ministries of Education (MoE) and of Health (MoH), the United Nations Population Fund (UNFPA), the United Nations Children’s Fund (UNICEF), the Swiss Development Cooperation (SDC) and the World Health Organization (WHO). To assess the compliance to the established quality standards for YFHS an external quality assessment using semi-structured questionnaires, client exit interviews and observation tools based on WHO’s Quality Assessment Guidebook [9] was carried out in a purposive sample of six YFHCs. Characteristics of the sample are summarized in Table 1. Individual interviews with adolescent clients and two focus group discussions were conducted. The two focus group discussions were carried out at the Neovita Centre in Chisinau (resource centre of the project) to learn about the perceptions of adolescents from the general adolescent populations and from a group with a more vulnerable background. Group 1 consisted of 10 boys and 7 girls, 15 years old, all from one school in the catchment area, different classes. Group 2: 3 girls 15–17 years old with histories of gender-based/domestic violence living in a temporary placement centre accompanied by their social worker (who after a while left).

**Table 1:** Characteristics of the six YFHCs included in the quality assessment

	Location (smaller town/bigger city)	Catchment population (10 – 24 years)	Included in economic analysis	Established in	Adherence to standards in the 2009 baseline survey	Included in coverage survey (2012)
1	Bigger city	28,000 (Focus on work with MARA)	Yes	2005	Well-performing	No
2	Smaller town	16,000	Yes	2006	Well-performing	Yes
3	Smaller town	20,000	No	2005	Performing less well	No
4	Smaller town	25,000	No	January 2013	N/A	No
5	Smaller town	19,916	No	January 2013	N/A	No
6	Smaller town	19,000	No	2012	N/A	Yes

The collected data was supplemented with data derived from a self-assessment that all YFHC were required to carry out by the Ministry of Health. To estimate the coverage of services, secondary data from a coverage assessment carried out in 2012 as well as data provided by the Ministry of Health and the data collected by the centres themselves was used. Knowledge, Attitude and Practice (KAP) of Youth 15–24 years surveys (2006–2012), National Household surveys (2006, 2008, 2010, 2012), TransMonEE (UNICEF) 2013 Database and data from the National Bureau of Statistics were used to estimate outcome and impact. Results of the *Economic Analysis Of Youth Friendly Health Services in the Republic of Moldova* [10] commissioned by UNICEF in 2013 as well as information provided by staff of the National Health Insurance CNAM was used to review costs of the YFHS.

## Results

In the following section we report the evaluation results according to the framework used as outlined above (namely results in relation to inputs, processes, outputs, outcomes, and impact, and the cost of YFHS).

### Inputs

Have the plan and budget the potential to achieve the desired outcomes and have the desired impact? Was sustainability built into the design?

The review had three main findings – firstly the overall the plan of the *Healthy Generation Project* addresses relevant areas and has – if implemented accordingly- the potential to achieve the desired outcomes and impact; secondly sustainability was built into the project design and thirdly some objectives stated are not reflected in the activities.

1. The goal of the project is to improve the sexual and reproductive health of young men and women in Moldova (particularly those vulnerable and most at risk) through increasing the demand, access to and utilization of quality youth friendly services and health related education programmes. Relevant outputs and corresponding activities are outlined in three broad areas: a) to make available youth-friendly-services, b) to increase knowledge and demand of services through school interventions and c) to create a supportive environment by working with communities. Given resource limitations, our review focused on output a) making available youth-friendly-services, as good quality services are a sine-qua-non for the success of the other components.
2. Sustainability was built into the design of this health sector intervention as the YFHCs have been established within the existing infrastructure and services are covered under the National Health Insurance. The project budget seems appropriate for the implementation of the outlined project activities; however, the budget of the National Health Insurance does not fully cover the costs required to provide quality health services according to the specified service package (refer to Section on Cost).
3. The stated objective to meet the needs of vulnerable and most at risk youth are not reflected in the activities outlined in the plan.

**Fig. 5:**
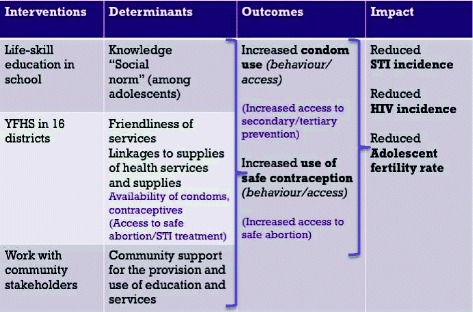
Implicit logic model of the Healthy Generation Project

No logic model showing the links between interventions, determinants, outcomes and impact was employed during the initial planning. The review team developed the logic model shown in Fig. 5, based on the Healthy Generation project’s plan. The interventions (namely: life skills education in schools, scaling up of YFHS and work with community stakeholders) aim at influencing the determinants affecting behaviours in relation to condom/contraception use as well as the accessibility of condoms and modern contraceptive methods. They also aim at increasing knowledge and influencing attitudes (and social norms) of adolescents as well as the availability and accessibility of YFHS and a supportive environment (community support of education and use of services). These factors could then lead to increased condom and contraception use and consequently to decreased HIV/STI infections and unintended adolescent pregnancies. Such a logic model points to important areas that are not included in the plan. For example, the determinants and outcomes that are currently not addressed are shown in violet. The current plan aims at primary prevention only – additional determinants and outcomes potentially being able to contribute to the desired impact are depicted in violet brackets.

### Process

Were the activities implemented according to plan?

The project worked effectively, carrying out the activities according to the plan. In the following section, the activities implemented in relation to each area for output a) *making YFHS available* are described briefly.

Horizontal scaling up actions:

1. The establishment of a YFHC in each district:
  Implementation targets were exceeded in relation to geographical scale up as 38 YFHC were established in 35 districts.
2. Capacity building of health care providers from YFHCs and primary health care services in adolescent health and quality improvement approaches:
  Stakeholders orientation meetings on the YFHS scaling up process were organized for district health leaders and district health management teams in 27 districts. In 16 of these 27 districts a total of 2500 persons were trained on adolescent health, trainees included YFHCs staff, family doctors, nurses and community resource persons.

Vertical scaling up actions:

1. The improvement of the regulatory basis for YFHS:
  The necessary legislation for the provision of youth friendly services has been partly put in place. Ministerial orders on job descriptions and staffing levels for YHFS have been formulated and released. At the time of the review YFHCs were not mandated to carry out medical interventions such as HIV testing and STI diagnosis and treatment and other treatments and therefore were not authorized to provide the full Package of Services as defined. A further barrier for the provision of services is that parental consent is required for some interventions, for example HIV testing in adolescents under the age of 18. Parental consent can however be waived if parents are abroad or otherwise not available.
2. The improvement of the financing mechanisms:
  YFHS are included in the National Health Insurance Scheme. This mainly covers staff costs and infrastructure. Costs of lab tests, gloves, examination tools (disposable specula etc.) and medications often need to be borne by the patient. Managers of YFHCs report that they try to find ways to make these consumables and medications available to patients who cannot afford them, however, there is no system in place to ensure that the complete package of services can be accessed free of charge in a systematic manner.
3. The improvement of the monitoring and evaluation (M&E) system:
  A Ministerial Order on monitoring youth friendly health services including patient record forms, monitoring forms and performance indicators to be piloted was released.
4. The revision of the medical university curricula to include adolescent health care:
  A post-graduate training course for service providers (in-service training as part of on-going education) has been developed, approved and integrated into the university curriculum for on-going medical education.

### Outputs

Were the implemented activities delivered with sufficient quality and coverage to achieve the desired outcomes and have the desired impact?

**Fig. 6:**
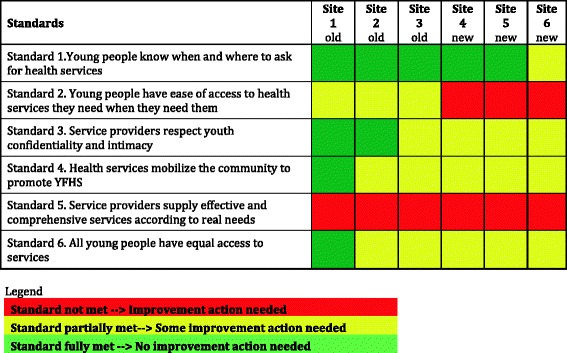
Performance of the six YFHCs in relation to the six quality standards in 2013

While impressive progress with geographical scale up (coverage) has taken place, service provision does not always meet the specified quality standards, thereby diminishing chances of having the desired impact and positively affecting national level impact indicators. Findings in relation to the quality of services included 1) scope for improvement in relation to almost all national quality standards (Fig. 1) [5] for the provision of YFHS (Fig. 6) in all six centres accessed and 2) a significant difference in the performance of the older versus the newer centres with the older centres performing consistently better.

1. The comprehensive *Package of Services* was not provided consistently in all the centres assessed (Standard 5, scored weakly already in the baseline assessment in 2009 as reported by Lesco G, Cebotari V. Baseline quality assessment of compliance of youth friendly health services in the Republic of Moldova to quality standards, 2009. Unpublished): neither HIV testing, emergency contraception, nor treatments for STIs or other diseases were found to be consistently available. Commodities and supplies were not always provided free of charge. Condoms and contraceptives were available in 5 of the 6 assessed YFHCs. In two (both of which were older centres) of the six centres condoms were available in the waiting area and could be taken by the adolescents without interaction with the doctor. Health staff of newer centres reported concerns regarding the use of modern contraceptives in adolescents: e.g., there was a widespread belief of health care providers that oral contraception is not safe for adolescents. Some of the centres seemed highly understaffed during the assessment; further they operate in small, cramped rooms within the general Family Medicine Centre. One centre cared for a large number of adolescents who are referred by the police and/or social workers, while the general adolescent population did not seem to access the centre.
2. There was a tangible difference in the quality of services provided to adolescents between the older NGO-born and subsequently Government designated centres and the centres that have been created recently by Ministerial Order. Older centres scored particularly higher in relation to acceptability i.e., standard 2 and 3. They displayed friendly surroundings, commitment and initiative to provide services to adolescents and young people. High satisfaction with services and youth friendly attitudes of staff were reported by clients for the older centres. Staff in these centres are clearly committed to the non-judgemental provision of services to young people and creating a welcoming atmosphere. The uptake of services by adolescents and young people in the older centres was higher. The newly established centres have created links with schools in their respective catchment areas as well as to other organizations that work with young people contributing to the achievement of Standard 1. There was a strong focus on information provision through schools and less focus on service provision and increase of the uptake of services by adolescents. Activities to mobilize the community to promote YFHS where found to be in line with the defined standard (Standard 4) in only one of the older centres, while in need of improvement in the remaining 5 centres that were assessed. Data on the access to services of young people in vulnerable situations was not consistently available; one of the older centres reported a large percentage of their clients belonging to vulnerable groups. All centres have policies for equal access in place (Standard 6).

The external review used secondary data from a coverage assessment carried out in 2012 as well as data provided by the Ministry of Health and the service utilization data collected by the centres to estimate the coverage of services. According to the YFHCs routine monitoring data, in 2012 a total 111,750 young people nationwide have benefited from any service or activity conducted by the YFHCs. This constitutes approximately 14 % of the total number of young people aged 10–24 years. A higher share of girls (61 %) compared to boys (39 %) have benefited from the services. The predominant age of visitors was 15–19 years (55 %), followed by 20–24 years (25 %) and lower proportions of 10–14 years old (10 %) and over 25 olds (10 %). The centres do not turn away the over 25 year olds although they do not belong to the target group. 38,745 young people or 35 % of the total, have come for services to the YFHCs, 49 % were reached with information sessions (mainly school based) and group activities (16 %). The 38,745 young people coming to the YFHCs received a total of 56,895 consultations or an average of 1.5 consultations per young person. Of the consultations, the majority 86 % were on health-related issues (while a smaller proportion of 14 % came to see the social worker). The most common reasons were related to prevention of unwanted pregnancies 21 %, management of STI 20 %, prevention and/or response to use of psychoactive drugs, including tobacco and alcohol 11 %, nutritional concerns and problems 9 %, concerns about puberty 8 %, mental health problems 8 %. Counseling for HIV accounted for 5 % and gender-based violence consultations represented 1 % of the total consultations provided. The remaining was reported under “other issues” and could not be specified more in detail.

At the time of the review no data was available on the number of especially vulnerable and most at risk adolescents (living on the street, abusing substances, engaged in unprotected sex) that received appropriate care and support provided by outreach workers (target 3000).

### Outcomes

Have the implemented activities lead to the desired outcomes i.e., improved knowledge of young people, behaviour changes e.g., increased use of condoms, contraception?

At the time of the review the desired outcomes had not been achieved yet.

The following two outcome indicators were chosen by the project, at the onset of the scaling up process, to measure progress:

1. Condom use by young people at last higher risk sex: proportion of young people 15–24 years of age who report using condoms at the last sexual intercourse with a non-regular partner (among both boys and girls) and
2. Current use of contraception by method and age group (% of 15–19 year olds and % of 20–24 year olds, group - married as well as sexually active unmarried women).

The baseline for the first indicator was 71 % (Youth Knowledge Attitude Practice on HIV, Survey, 2008) in 2008 with a target set for 2017 of 78 %. In 2012 74.9 % of young people 15–24 years of age reported using condoms at the last sexual intercourse with a non-regular partner (Youth knowledge Attitude Practice Survey on HIV, 2012).

For the second indicator the baseline was 7.5 % of 15–19 year olds and 29.8 % of 20–24 year olds in 2005 (Demographic and Health Surveys) with a target set of 10 and 35 % for 2017 respectively. No interim data are currently available.

### Impact

Did the achieved outcomes have the desired impact e.g., reduced adolescent pregnancies and reduced STI/HIV rates?

The following three impact indicators were chosen by the project, at the onset of the scaling up process to measure progress:

1. Sexually Transmitted Infection (STI) incidence among 15–19 year old adolescents (syphilis and gonorrhoea),
2. HIV incidence among young people (15–24 years) and
3. Adolescent fertility rate (in adolescent girls aged 15–19 years).

The baseline for the first indicator was 188.5/100,000 in 2008 with a target set of 156/100,000 for 2017. In 2011 the STI incidence among 15–19 year old adolescents was 172/100,000. For the second impact indicator the baseline was 16/100,000 in 2008 (Knowledge Attitude Practice Youth Survey on HIV 2008) with the target of 16/100,000 for 2017. Unpublished data provided by the MoH shows an HIV incidence for the 15–24 years age group of 18.4/100,000 for 2012. For the third impact indicator the baseline was 26/1000 in 2008 with a target of 22/1000 for 2017. The Adolescent fertility rate was 25.7/1000 in 2011.

### Cost

Were available resources used efficiently? Are the services sustainable?

The project used available resources efficiently and – given its integration into the health system within the existing infrastructure and coverage under the National Health Insurance - services are likely to be sustained beyond the end of the project. The budget provided by the National Health Insurance does not however cover the full costs required to provide quality health services according to the specified service package: The established facilities are located within the premises of the existing infrastructure of the health system and are covered under the National Insurance Scheme, which facilitates the sustainable existence of YFHS centres. The National Insurance Company allocated 5,100,000 MDL^1^ for the Youth Friendly Health Services in 2012 that are currently allocated on a per capita system, thus the facilities catering for a larger district/catchment area are receiving more funds.

In addition it has to be noted that the current allocation of funds falls short (by 44 %) vis-à-vis the funding needs for good quality YFHS as estimated in the Economic analysis, namely 9,100,000 MDL^2^ for 28 centres and by 57 % if extrapolated to funding needs of all established 37 centres (9 addition YFHC times 300,000 MDL^3^ (needed for good quality services = 2,700,000 MDL^4^ to cover costs of 9 additional centres).

Limitations:

Firstly, we found the evaluation framework useful to structure the review and subsequent planning of further activities to address identified limitations. Its use allowed us to identify issues that they might have gone unnoticed with a less-structured approach. It also helped in promoting a shared understanding of the prerequisites for successful implementation of health interventions for adolescents. While a systematic use of a logical framework would be preferable from the outset of the health service intervention, doing so even at the evaluation stage is useful.

Secondly, data used in the analysis e.g., on service utilization and on behavioural and health outcomes were drawn from secondary sources. Data quality and data match between geographic areas/periods of implementation and results was sometime poor.

## Discussion

The principal findings of the review were as follows:

- Plan: The goal of the project is clear. The project’s planned activities and anticipated outputs are aligned to its goals. They been adequately resourced. If implemented with adequate quality and coverage, the planned package of activities has the potential to achieve the aimed for behavioural outcomes and health impact. However, there is one important exception – there are no activities planned to address young people who are most vulnerable and most at risk. Sustainability was adequately built into the design. The project aimed to improve the quality of health services provided by existing health workers through existing health facilities and systems.
- Process (implementation of plan): The project carried out activities in line with the plan where it had the authority and ability to do so e.g., meeting stakeholders, training health care professionals and carrying out follow up. However, project’s activities were slowed/hindered by factors beyond its control notably weaknesses in the regulations, inadequate financial allocation in the health insurance system, and delays in putting a monitoring system in place.
- Outputs (quality and coverage): While there were impressive increases in geographic coverage, health services were not delivered with adequate quality especially in the newly established health facilities. Data from the coverage assessment and from service utilization data gathered by health facilities suggest that while health service utilization was not small (14 % of 10–24 year olds), many adolescents and young people have not obtained services. Further, we do not know which groups of adolescents have received services and which groups have not done so.
- Behavioural outcomes: Data from nationwide surveys indicate that there has been a slight increase in 15–24 year olds who report condom use at the last sexual intercourse with a non-regular partner. There is no data on the use of contraception.
- Health impact: Again from nationwide surveys, there is evidence of some decline in STI incidence and fertility in 15–19 year-old adolescents, and of a slight increase in HIV incidence in 15–24 year olds.
- Cost: The funds made available by the National Insurance Company for YFHS in 2012 did not fully cover the costs of provide YFHS as calculated in the Economic Analysis. Firstly, the Economic analysis did not take into account out-of-pocket expenses for the purchase of supplies needed for examination (e.g., gloves) and medication. Secondly, the Economic analysis was based on the services that were being provided not on the ones that are required to be provided. Further, funds were allocated on a per capita system whereby the facilities catering for a larger district/catchment area received more funds than others.
- Sustainability: The Government of Moldova is committed to sustaining the scale up of health service provision to adolescents and young people, and has taken steps that relate to the vertical scaling up component of the WHO-ExpandNet framework. These steps include the stipulation of national quality standards for health service provision to adolescents, the deployment of staff, the assignation of health facilities and the inclusion of activities relating to YFHS in district level (i.e., rayonal work plans) in addition the government acted immediately on the findings of the external review changing policies and regulations that acted as barriers to the provision of the required package of health services at the youth-friendly health facilities.

The project has a sound design and has been implemented energetically. Because of pressure to expand the geographic focus beyond what was originally planned, its efforts were dispersed. The project was unable to give the new centers the additional support they need. This can explain why their improvements in quality have been less than expected. The slight declines in STI incidence and fertility in adolescents may be caused by programming efforts over the years and not because of this project per se. Legal and policy barriers hindered improvements in quality as well. Designating health facilities to be made youth friendly and assigning health workers to manage them can be done fairly quickly, improving performance takes time and effort. Given that the evaluation was carried out at the end of phase 1 of the project and some facilities were only recently designated as youth friendly health centres it may have been to early to see an increase in quality. As adolescent users experience improved service quality and their peers hear about this from them, utilization will improve in time.

Assessments, reviews and evaluations in other countries have shown that well-designed and well-conducted efforts can lead to improvements in the quality of health service provision [11]. When combined with complementary interventions to improve knowledge and understanding, promoting the adoption of healthy behaviours and creating an enabling environment for this to happen, improvements in the quality of health service provision can lead to increases in health service utilization and in uptake of health interventions e.g., contraception and HIV testing and counselling [12]. Other assessments, reviews and evaluations have most commonly examined the outputs of efforts to make health services youth friendly (quality and coverage), behavioural outcomes (e.g., use of services and uptake of contraception) and rarely health impacts (e.g. HIV incidence). Very few have carefully examined inputs and processes as this review has done [13, 14].

Our review has pointed to areas of strength and to areas of weakness. It also pointed to corrective actions that are needed as the project moves to the next phase. These are summarized below:

- Policies and regulations that act as barriers to the provision of the required package of health services (e.g., screening, diagnosis and treatment of STI) should be removed. This has already been addressed following the results of the review.
- Improve the knowledge, understanding and motivation of health workers in the newly designated YFHC to address the problem of poor quality, using approaches that go beyond training such as collaborative learning and job shadowing which have been included in the next phase for the project.
- Strengthen data collection or service utilization in all YFHC.
- Ensure that alongside efforts to improve the quality and coverage of health service provision, efforts are made to improve the provision of information and education, and to provide a safe environment within and beyond the health sector in a time of economic constraints and rapid social change.
- Ensure that the National Health Insurance Company allocates adequate resources for YFHS provision, by combining the prospective per capita funding system which is currently in place with a retrospective supplementary payments if specified quality indicators are met and the number of patients treated.
- Step up effort to reach adolescents who are at heightened vulnerability and risk.

The macroeconomic situation in Moldova and the financial constraints in which the health sector is currently operating will most likely continue to pose challenges to the provision of youth friendly health services in the future. Additional external support will be needed until at least the provision of the package of services free of charge for all adolescent (or at a minimum for the most vulnerable adolescents) is consistently being implemented and becoming part of the established practice.

## Conclusion

The Healthy Generation project was well designed and energetically implemented in line with the plan. It has contributed to tangible improvements in the quality of health service provision, and to their uptake. While progress has been made, considerable work is needed, especially in the newer centers. If the efforts of the Healthy Generation project are stepped up, if weaknesses in its planning and implementation are adressed, if complementary activities to build knowledge, understanding, skills and an enabling environment are carried out, the project can be expected to improving the health and well being of Moldova’s adolescents.

## Footnotes

1 5,100,000 MDL = USD 374,194 (1 US Dollar (USD) = 13.6293 Moldovan Leu (MDL) (13 Jun 2014))

2 9,100,000 MDL = USD 667,679

3 300,000 MDL = USD 22,011

4 2,700,000 MDL = USD 198,103
